# Rifaximin as a Potential Treatment for IgA Nephropathy in a Humanized Mice Model

**DOI:** 10.3390/jpm11040309

**Published:** 2021-04-16

**Authors:** Vincenzo Di Leo, Patrick J. Gleeson, Fabio Sallustio, Carine Bounaix, Jennifer Da Silva, Gesualdo Loreto, Sanae Ben Mkaddem, Renato C. Monteiro

**Affiliations:** 1 INSERM U1149, Centre de Recherche sur l’Inflammation, 75018 Paris, France; vincenzodileo88@yahoo.it (V.D.L.); james.gleeson@inserm.fr (P.J.G.); bounaix.carine@gmail.com (C.B.); jennifer.da-silva@inserm.fr (J.D.S.); 2 CNRS ERL8252, 75018 Paris, France; 3 Faculté de Médecine, Université Paris Diderot, Sorbonne Paris Cité, Site Xavier Bichat, 75018 Paris, France; 4 Inflamex Laboratory of Excellence, 75018 Paris, France; 5 Division of Nephrology, Dialysis, and Transplantation, Department of Emergency and Organ Transplantation, University of Bari, 70124 Bari, Italy; fabio.sallustio@uniba.it; 6 Service d’Immunologie, DHU Fire, Assistance Publique de Paris, Hôpital Bichat-Claude Bernard, 75018 Paris, France

**Keywords:** IgA Nephropathy, rifaximin, microbiota, α1^KI^-CD89^Tg^ mice

## Abstract

IgA Nephropathy (IgAN) is the most common glomerulonephritis worldwide, characterized by the mesangial deposition of abnormally glycosylated IgA1 (Gd-IgA). The production of Gd-IgA occurs in mucose-associated lymphoid tissue (MALT). The microbiota plays a role in MALT modulation. Rifaximin (NORMIX^®^), a non-absorbable oral antibiotic, induces positive modulation of the gut microbiota, favoring the growth of bacteria beneficial to the host. Here, we evaluate the effect of rifaximin on a humanized mice model of IgAN (α1^KI^-CD89^Tg^). **Methods:** The α1^KI^-CD89^Tg^ mice were treated by the vehicle (olive oil) or rifaximin (NORMIX^®^). Serum levels of hIgA, hIgA1–sCD89, and mIgG–hIgA1 immune complexes were determined. Glomerular hIgA1 deposit and CD11b+ cells recruitment were revealed using confocal microscopy. Furthermore, the mRNA of the B-Cell Activating Factor (BAFF), polymeric immunoglobulin receptor (pIgR), and Tumor Necrosing Factor-α (TNF-α) in gut samples were detected by qPCR. **Results:** Rifaximin treatment decreased the urinary protein-to-creatinine ratio, serum levels of hIgA1–sCD89 and mIgG–hIgA1 complexes, hIgA1 glomerular deposition, and CD11b+ cell infiltration. Moreover, rifaximin treatment decreased significantly BAFF, pIgR, and TNF-α mRNA expression. **Conclusions:** Rifaximin decreased the IgAN symptoms observed in α1^KI^-CD89^Tg^ mice, suggesting a possible role for it in the treatment of the disease.

## 1. Introduction

IgA Nephropathy (IgAN) is a frequent cause of end-stage renal failure (about 20–40% of cases) [1] and it is characterized by dominant mesangial IgA deposition [2]. Microscopic hematuria and proteinuria are the most common clinical presentations [3]. Abnormally glycosylated IgA1 (Gd-IgA1) has a central role in the multi-hit process in IgAN patients [4]. Moreover, it has been demonstrated that there are two IgA receptors involved in IgAN pathogenicity: the FcαRI (CD89), expressed by blood myeloid cells and the transferrin receptor (CD71), expressed by mesangial cells [5]. Gd-IgA1-CD89 interaction induces the release of the extracellular portion of CD89 (soluble form of CD89) leading to the formation of circulating CD89-IgA immune complexes, which bind to CD71 leading to IgA1 deposits and mesangial cells proliferation in IgAN patients [6]. In patients with progressive disease, the IgA-CD89 complex has a role in the pathogenesis of IgAN and it seems to be positively correlated with proteinuria, microalbuminuria, and with some features of the Oxford score (endocapillary and extracapillary proliferation) [7].

Moreover, genetic variants, lifestyle, diet, and environmental factors contribute to disease onset [8]. The mucose-associated lymphoid tissue (MALT) is largely involved in the pathogenesis of the disease and, considering that it is influenced by antigenic stimulation from the commensal microflora, in recent years, scientific efforts have focused on the possible role of the microbiota and its modulation on the development and progression of IgAN [9]. Using antibiotics to manipulate the gut microbiota may represent a potentially effective treatment option for IgAN. A previous study by Chemouny et al. [10] has demonstrated that antibiotic treatment (ampicillin, vancomycin, neomycin, and metronidazole) of an IgAN mice model (α1^KI^-CD89^Tg^ mice) reverses the IgAN phenotype without affecting serum IgA levels.

Rifaximin is a non-absorbable oral antibiotic that inhibits the synthesis of bacterial RNA by binding the β subunit of bacterial DNA-dependent RNA polymerase. It demonstrates bactericidal and bacteriostatic activity against both Gram-positive and Gram-negative aerobic and anaerobic bacteria. It has been proven to be safe and well-tolerated. Previous studies have shown that rifaximin can alter intestinal flora, inhibit bacterial attachment, prevent intestinal inflammation, and modulate gut barrier function [11]. This special feature distinguishes rifaximin from other systemic antibiotics. However, it is not clear whether orally administered rifaximin can prevent the development of IgAN by down-regulation of the inflammatory response triggered by gut microbes.

In this study, we investigated the effect of rifaximin on the IgAN progression, using a humanized mouse model of IgAN (α1KI-CD89^Tg^ mice). Rifaximin decreased the IgAN phenotype in a humanized mouse model of IgAN opening new therapeutic avenues for this disease.

## 2. Materials and Methods

### 2.1. In Vivo Experiments

Twelve-week-old α1^KI^-CD89^Tg^ mice (n = 24) were raised and maintained in a specific pathogen-free mouse facility at the Centre for Research on Inflammation, Paris, France. All experiments were performed in accordance with the National Ethics Guidelines and with the approval of the Local Ethics Committee. These 12-week-old mice were divided into two groups to receive, by oral gavage, olive oil (n = 12) or rifaximin (NORMIX^®^) 100 mg/kg/die dissolved in olive oil (n = 12) [APAFIS number: #14265] for two weeks. We used olive oil because rifaximin is water-insoluble.

Urine was collected before starting, every four days, and at the end of the treatment experiment. Blood was collected by retro-orbital bleeding and the mice were sacrificed by cervical dislocation; the blood samples were centrifugated at 1500× *g* rpm for 10 min at room temperature. The serum was collected and kept frozen at −80 °C until use. Kidneys and part of the ileum (2 cm above the ileocecal valve) were collected. Organs were conserved in OCT (CellPath Ltd., Newtown, Powys, UK).

### 2.2. Histopathology Procedures

For immunohistochemistry, 4 µm sections of cryostat frozen kidney were fixed in acetone for 30 min. Immunofluorescence staining was performed with goat anti-hIgA FITC (1/50, Southern Biotech, Birmingham, AL, USA) and Phalloidin (1/100, Invitrogen, Carlsbad, CA, USA) or anti-mouse CD11b antibody (M1/70) FITC (1/100, Abcam, Cambridge, UK) and Phalloidin (1/100, Invitrogen). Slides were mounted with Immuno-mount (Thermo Scientific, Waltham, MA, USA) and read with an immunofluorescent microscope (Zeiss, Oberkochen, Germany, LSM 780). Mean fluorescence intensity area positive for hIgA1 or for CD11b was measured using ImageJ and it was normalized for the total glomerular area.

### 2.3. Enzyme-Linked Immunosorbent Assay

Serum levels of hIgA were determined with a sandwich enzyme-linked immunosorbent assay (ELISA). Goat anti-hIgA (Bethyl Laboratories, Montgomery, TX, USA, A80–120A, 1:500 dilution) was used for coating. Sera (1:3000 diluted) were then added and revealed with goat anti-hIgA antibody HRP conjugated (Bethyl Laboratories, A80–120P, 1:50,000 dilution). The optic density (OD) was measured at 450 nm.

The hIgA1–sCD89 and mIgG–hIgA1 complexes were determined with ELISA [12]. A3 mAb anti-human CD89 (5 µg/mL, homemade [13]) or goat anti-hIgA (Bethyl Laboratories, 1:500 diluted) were used for coating.

Sera (1:10 diluted) were then added and revealed with goat anti-hIgA (Southern Biotech, 1:2000 dilution) or goat anti-mouse IgG (Southern Biotech, 1:5000 dilution) coupled with alkaline phosphatase (Southern Biotech, Birmingham, AL, USA). The OD at 405 nm was measured after 4 h from the addition of alkaline phosphatase substrate (Sigma-Aldrich, St. Louis, MO, USA). The complex levels were expressed as OD.

### 2.4. Real-Time PCR

Total RNA from mouse small intestine (four mice for each group) was isolated with RNABle (Eurobio laboratories, Les Ulis, France), according to the manufacturer’s instructions, and complementary DNA was synthesized using Moloney-Murine Leukemia Virus reverse transcriptase (M-MLV RT, Invitrogen). cDNA was subjected to quantitative real-time PCR using a Chromo4 Real-Time PCR Detection System (Bio-Rad Laboratories, Marnes-la-Coquette, France). The mouse TNF-α, pIgR, BAFF, and ß-actin primers used and the corresponding Taqman probes are listed in Table S1 (Supplementary data 1).

The data from the qPCR were converted to 2-Ct, where Ct represents the threshold cycle. The mean Ct value of the duplicate PCRs was determined, and the mean 2-ΔΔCt was calculated from the duplicate cDNAs. PCR data were reported as the relative increase in mRNA transcripts versus that found in the pool of RNA of olive-oil-treated mice and corrected using the respective levels of ß-actin mRNA.

### 2.5. Statistical Analysis

Statistical analyses were performed with GraphPad Prism 6.0 (GraphPad Software, Inc., San Diego, CA, USA). We compared the results of the treatment and control group using the Mann–Whitney U test. The qPCR data were reported as the relative increase in mRNA transcripts versus that found in respective tissues from vehicle mice, corrected by the respective levels of β-actin mRNA, used as an internal standard. All the values of olive-oil-tested mice are 1. Statistical analyses were performed using the Wilcoxon test. Differences between groups were considered to be significant at a *p*-value of <0.05.

## 3. Results

### 3.1. Rifaximin Reduces the Disease Phenotype in IgAN Mice Model

**Figure 1:**
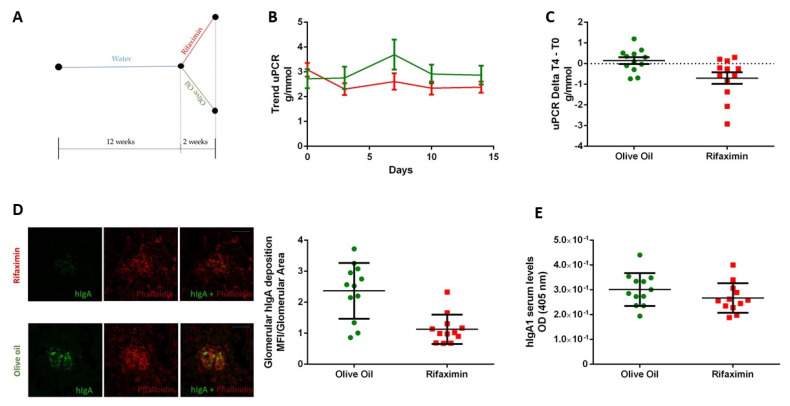
(**A**) Twelve-week-old mice were given 100 mg/kg of Rifaximin or of olive oil by oral gavage for two weeks before sacrifice. (**B**) Trend of uPCR from 12 weeks to sacrifice. (**C**) uPCR Delta t3-t0, where t3 is the uPCR after two weeks of treatment and t0 is uPCR before starting antibiotic or vehicle. (**D**) Representative sections of kidneys after immunostaining with anti-hIgA-FITC antibody and Phalloidin–Alexaflour 564 to underline glomerular structures (green anti-hIgA1, red phalloidin) and the ratio between the glomerular area positive for hIgA1 and total area of the glomerulus, measured using ImageJ. (**E**) hIgA serum level measured by ELISA in mice that received antibiotics or vehicle and compared using the Mann–Whitney test.

Twelve-week-old α1KI-CD89Tg mice spontaneously present mesangial hIgA1 deposition, associated with proteinuria, mimicking IgAN in humans as described previously [13]. Mice treated with rifaximin for two weeks had a reduction in proteinuria (initial and final uPCR mean: 3.09 g/mmol and 2.39 g/mmol, respectively) compared to the mice treated with just the vehicle (olive oil) which showed an increase in proteinuria (initial and final uPCR mean in the oil group: 2.72 g/mmol and 2.87 g/mmol, respectively). There was no statistically significant difference in uPCR between the groups at T0 (*p* > 0,05; Figure 1B), while we found a significant difference between uPCR at T0 and uPCR after 14 days [delta T4–T0 (p* = 0.0172; Figure 1C)]. Moreover, anti-hIgA immunostaining of mouse kidneys revealed that hIgA1 deposition was significantly reduced in antibiotic mice compared to the olive oil group (p** = 0.0014, Figure 1D). To explore whether rifaximin affects the level of the total circulating hIgA1, we measured the serum IgA1 level by ELISA. Serum levels of hIgA1 were similar in the rifaximin group and the vehicle group (*p* > 0.05, Figure 1E).

**Figure 2:**
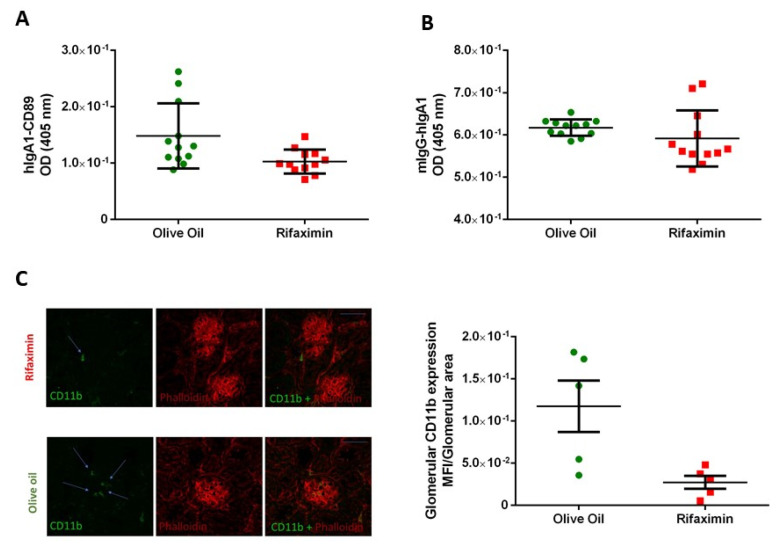
Antibiotic treatment decreases the formation of hIgA1–sCD89 and of mIgG–hIgA1 complexes but not the hIgA1 serum level. (**A**) hIgA-CD89 complexes in mice that received rifaximin or olive oil. (**B**) Levels of mIgG-hIgA complexes in mice that received antibiotics or vehicle. Anti-hIgA or A3 monoclonal-antibody anti-human CD89 was used for coating. Polyethylene glycol precipitated sera were then added; detection with anti-hIgG or anti-hIgA-HRP. Serum hIgA1 and mIgG levels were measured by ELISA. Statistical analyses were performed using Mann–Whitney test. (**C**) Quantification of glomerular cells was performed by counting the area positive for CD11b measured using ImageJ. Representative sections of kidneys after immunostaining with anti-CD11b-FITC antibody and Phalloidin–Alexaflour 564 to underline glomerular structures (green anti CD11b, red phalloidin) and the ratio between the glomerular fluorescence area positive for CD11b and total area of the glomerulus, measured using ImageJ.

In contrast, mice in the antibiotic group showed less hIgA1-CD89 (Figure 2A, p* = 0.0145) and mIgG-hIgA1 complexes (Figure 2B, p* = 0.0447) than the control group. To evaluate the effect of rifaximin on kidney inflammation in the IgAN mice model, we assessed the immunofluorescence to analyze whether rifaximin affects CD11b+ renal infiltration. The antibiotic reduced the development of glomerular inflammation as illustrated by less CD11b-positive area normalized for the total glomerular area (Figure 2C, p* = 0.0317).

### 3.2. Rifaximin Group Showed Less TNF-α, BAFF, and pIgR mRNA Gut Expression Levels

**Figure 3:**
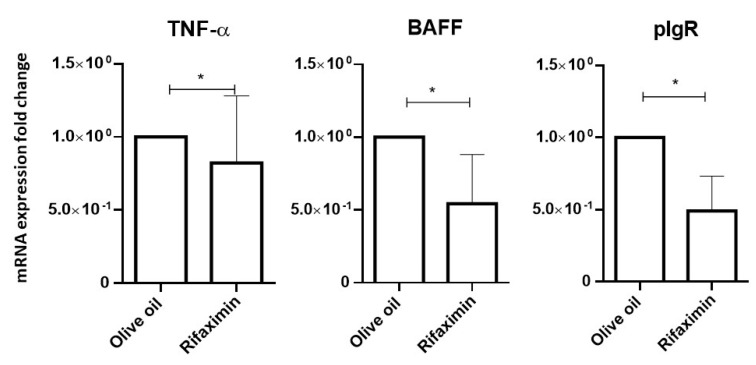
Antibiotic treatment reduced the expression of TNF-α, BAFF, and pIgR mRNA in small intestine samples, compared with the control. The qPCR data were reported as the relative increase in mRNA transcripts versus that found in respective tissues from vehicle mice, corrected by the respective levels of β-actin mRNA, used as an internal standard. All the values of olive oil tested mice were 1. Statistical analyses performed using the Wilcoxon test.

It has been shown that epithelial-derived BAFF is the major modulator of B cell development and it has a key role in IgA class switching and plasma cell survival in the MALT [14,15]. Consistent with the effect of rifaximin on renal inflammation, mice treated with this antibiotic present a significant decrease of TNF-α, BAFF, and pIgR mRNA gut expression when compared to the control group (respectively p* = 0.0369, p* = 0.0490, p* = 0.0271). TNF-α, BAFF, and pIgR mRNA expression levels are illustrated in Figure 3.

## 4. Discussion

Although IgAN seems to be a final common endpoint of different pathological processes, numerous studies indicate that it is closely associated with perturbed homeostasis of intestinal-activated B cells and intestinal IgA class switch and, at the same time, with alterations of the gut microbiota and of intestinal-barrier, in humans and animal models [9,16,17].

The intestinal-activated B cells play a central role in pathogens and mucosal inflammatory diseases [18,19]. Epithelium-derived BAFF is the major modulator of B cell development and it has a key role in IgA class switching and plasma cell survival in the MALT. Moreover, the gut microbiota, through toll-like receptor (TLR) ligation on mucosal dendritic cells, can induce inflammation and production of proinflammatory cytokines, inducing the overexpression of BAFF mRNA in mucosal epithelial cells [20]. The upregulation of BAFF is associated with hyper-IgA syndrome in the gut and the deposition of IgA immune complexes in the glomerular mesangium [16]. Given these findings, over the last few years, the need to test new interventions in IgAN patients and new therapeutic strategies, such as the administration of antibiotics or dietary implementation with prebiotics and/or probiotics, or through fecal microbiota transplantation (FMT) [21,22], has earned high demand, especially following the latest results from trials of gut-targeted corticosteroids [23,24].

Here we investigated the effects of rifaximin, a broad-spectrum, non-absorbable, oral antibiotic, on α1KI-CD89Tg mice. Rifaximin inhibits microbe-induced immune response, acts on intestinal barrier integrity, and has a direct anti-inflammatory property through binding to the Pregnane X Receptor (PXR) and modulating gut microbiota [11,25,26,27]. In particular, it is already demonstrated that rifaximin increases the relative abundance of beneficial intestinal bacteria, such as *Lactobacillus* and *Bifidobacterium* [28], reduces activation of T helper 17 cells [29], and attenuates TLR-4/NF-kB pathway activation in the gut [30].

However, the therapeutic effect of rifaximin has not yet been studied in IgAN. In this study, through a combination of ELISAs, confocal microscopy, and qPCR, we analyzed the characteristic features of α1KI-CD89Tg mice and the impact of rifaximin.

Our hypothesis is that, in IgAN, the leaky gut syndrome and the dysbiosis can lead, through the production of pro-inflammatory cytokines and an increased bacterial translocation, to gut inflammation, activation of dendritic cells (DCs) and, via T-cell-independent pathway (BAFF mediated), overproduction of Gd-IgA1 [14]. These are secreted, in the form of IgA1 dimers, across intestinal epithelial cells by transcytosis, in which pIgR facilitates the release of secretory IgA (sIgA) into the gut lumen. Rifaximin, through restoring symbiosis (including increased *Bacteroidetes/Firmicutes* ratio, as well as selective promotion of probiotic populations) and by binding PXR, is able to restore intestinal barrier function and inhibit the TLR-4/NF-kB signaling pathway in the small intestine [30], leading to decreased TNF-α synthesis [26]. Since the expression of BAFF and pIgR genes is regulated by TNF-α [31,32], the reduction of the latter causes the down-regulation of pIgR, BAFF and, consequently, of Gd-IgA1 (the proposal mechanism of rifaximin action in IgAN is represented in Supplementary data 2). Indeed, under gene expression profiling, our findings support reduced gut inflammation following rifaximin treatment, showed by a downregulation of TNF-α and BAFF gene transcription. Moreover, although we did not find any difference in IgA serum levels between the two groups, we found a reduction of hIgA1–mIgG, hIgA1–sCD89 complexes serum levels (the main serum markers of disease in this animal model), and of IgA mesangial deposition that could be explained by a greater availability and ability of IgA to bind CD89 or mIgG or mesangial cells in the control group compared to the treated group.

Although the “eubiotic” effect of rifaximin on gut microbiota is established [27], the exact mechanism of action in IgAN requires further investigations. Indeed, there were some limitations to this study, particularly the lack of the analysis of microbiota, that did not allow us to state whether our results are due to the modulation of the intestinal microbiota or if they are due to other effects of rifaximin on the gut.

In conclusion, the present study demonstrated that rifaximin reduces the progression and the severity of IgAN observed in humanized mice (α1^KI^-CD89^Tg^) and showed that rifaximin might open a new therapeutic avenue for IgAN. However, more detailed research is required to establish the precise molecular mechanism involved and the exact role of microbiota in this pathway.

## Data Availability

Data is contained within the article.

## Supplementary Materials

The following are available online at https://www.mdpi.com/article/10.3390/jpm11040309/s1, Supplementary data 1 Table S1; Supplementary data 2 Proposal mechanism.
