# Supplementary material for: Rifaximin as a Potential Treatment for IgA Nephropathy in a Humanized Mice Model
Source: J Pers Med. 2021 Apr 16;11(4):309. doi: 10.3390/jpm11040309 (PMC8072762; doi:10.3390/jpm11040309)
Supplement: Supplementary file 1 [file jpm-11-00309-s001.zip › jpm-1136518-supplementary.pdf]

Supplementary data 1

Table 1

Gene	Accession number	5’-3’ sequence	Origin
ß-actin (F)	(NM_007393)	5’AGAGGGAAATCGTGCGTGAC -3’	Eurofin
ß-actin (R)	(NM_007393)	5’CAATAGTGATGACCTGGCCGT-3’	Eurofin
ß-actin (P)	(NM_007393)	5’CACTGCCGCATCCTCTTCCTCCC-3’	Eurofin
TNF-α (F)	(NM_013693)	5′CATCTTCTCAAAATTCGAGTGACAA-3′	Eurofin
TNF-α (R)	(NM_013693)	5′TGGGAGTAGACAAGGTACAACCC-3′	Eurofin
TNF-α (P)	(NM_013693)	5′CACGTCGTAGCAAACCACCAAGTGGA-3′	Eurofin
pIgR	(NC_000067.6)	Pre-design primer qMmuCIP0028503	Bio-Rad
BAFF	(NC_000074.6)	Pre-design primer qMmuCIP0032122	Bio-Rad

F: Forward; R: Reverse; P: Probe (FAM)

Supplementary data 2

Proposal mechanism

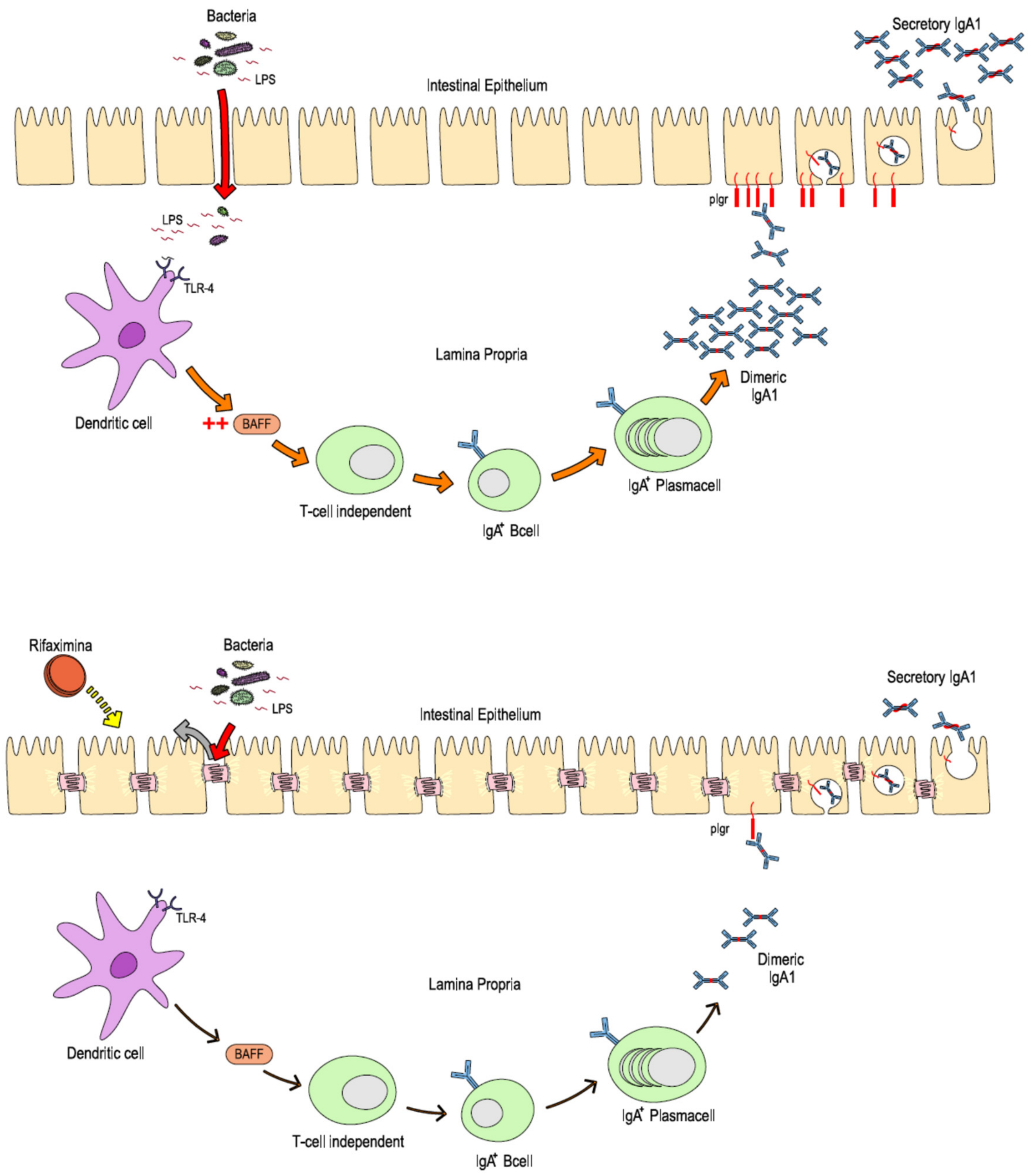

Proposal mechanism: **rifaximin inhibits microbes-induced immune response, acts on intestinal barrier integrity and has an anti-inflammatory property through the binding to Pregnane X Receptor (PXR) and modulating gut microbiota [11, 24-26]. Its binding to PXR, is able to restore intestinal barrier function and to drop the TLR-4/NF-kB signaling pathway activation in the small intestine, leading to an inhibition of TNF-α synthesis. The reduction of the latter causes the upregulation of tight junction expression and the down synthesis of pIgR, BAFF and, consequently, of Gd-IgA1.**
